# *Prunus spinosa* L. Branches as a New Source of Condensed Tannins: Phytochemical Profile and Antioxidant, Cytotoxic and Genotoxic In Vitro Evaluation

**DOI:** 10.3390/antiox14121408

**Published:** 2025-11-26

**Authors:** Oana Teodora Apreutesei, Carmen Elena Tebrencu, Daniela Gherghel, Lăcrămioara Anca Oprică, Irina Volf, Gabriela Vochița

**Affiliations:** 1 Stejarul Research Centre for Biological Sciences, National Institute of Research and Development for Biological Sciences, Alexandru cel Bun Street, 6, 610004 Piatra Neamt, Romania; oana.apreutesei@incdsb.ro; 2 Research and Processing Centre for Medicinal Plants PLANTAVOREL S.A., 46, Cuza Voda Street, 610019 Piatra Neamţ, Romania; carmen@plantavorel.ro; 3 Academy of Romanian Scientists, 3 Ilfov, 050044 Bucharest, Romania; 4 Institute of Biological Research Iasi, Branch of NIRDBS—National Institute of Research and Development for Biological Sciences, 47, Lascar Catargi Street, 700107 Iasi, Romania; daniela.gherghel@icbiasi.ro (D.G.); gabriela.vochita@icbiasi.ro (G.V.); 5 Faculty of Biology, Alexandru Ioan Cuza University, 20A Carol I Bd., 700506 Iasi, Romania; lacramioara.oprica@uaic.ro; 6 Faculty of Chemical Engineering and Environmental Protection, Gheorghe Asachi Technical University of Iași, 73, Prof. Dr. Docent D. Mangeron Street, 700050 Iași, Romania

**Keywords:** *Prunus spinosa* L., waste biomass, condensed tannins, antioxidant, cytotoxic and genotoxic activity

## Abstract

(1) Background: *Prunus spinosa* L. is known for its polyphenolic profile, including condensed tannins, compounds associated with various biological activities, including antiproliferative effects. Its woody biomass, such as branches, remains largely underexplored, as a few studies have investigated its potential bioactive content. This study aimed to characterize and evaluate the biological potential of crude extract (PS) obtained from *P. spinosa* branches. (2) Methods: The extract (PS) was prepared using microwave-assisted extraction (MAE) under optimized green conditions (70% ethanol, 1/10 solid–liquid ratio, 5 min, 600 W). Its chemical profile was analyzed by High-Performance Thin-Layer Chromatography (HPTLC). Antioxidant capacity was assessed through HPTLC-DPPH, DPPH and ABTS assays. In vitro cytotoxicity and genotoxicity were evaluated on HeLa (tumoral) and Vero (normal) cell lines using MTT and Comet assays. (3) Results: HPTLC analysis revealed the presence of condensed tannins. The extract demonstrated potent radical scavenging activity (IC_50_ 1.02 ± 0.25 mg/mL), dose-dependent cytotoxicity, and higher sensitivity of HeLa cells. Genotoxic effects were significantly more pronounced in tumor cells than in normal ones. (4) Conclusions: These findings highlight the condensed tannins’ phytochemical profile, antioxidant and selective antitumor properties of PS, supporting its valorization as a sustainable source of multifunctional bioactive compounds.

## 1. Introduction

In the growing context of a green and circular economy, gaining knowledge about the composition of each biomass source is crucial, as this enables their full and sustainable exploitation. A wide variety of bioresources are distributed across the globe, playing an important role in human survival. Many studies on biomass resources have focused on the production of high-value products and the evaluation of their biological potential. In recent years, the bioactivity of natural products in relation to human health, pharmaceutical applications, nutrition, and therapy has been the subject of extensive research [1,2,3,4].

Medicinal plants, as primary sources of natural bioactive compounds, are receiving increasing attention as constituents of functional products active in the prevention and adjuvant therapy of many chronic diseases [5], while numerous botanical sources have been explored as promising candidates for anticancer therapy. Further research is needed to turn this potential and promising role of plants into a viable form for developing treatment options with minimal or no side effects [6]. Plant-based compounds such as tannins, flavonoids, terpenoids, and steroids have attracted scientific interest in recent years due to their diverse pharmacological effects, including antioxidant, anticancer, and antimicrobial activities [7,8,9,10,11].

Polyphenols are classified as secondary metabolites with well-documented dietary antioxidant benefits for human health. Tannins represent an important class of polyphenols often neglected. Condensed tannins have been studied extensively as potential defenses against pathogens or for their beneficial effects, such as antioxidants for human health [12].

Tannins are widely distributed in the plant, being found especially in herbaceous species, shrubs, cereals and medicinal plants [13]. Furthermore, they have been identified in some fruits, such as bananas, blackberries, apples and grapes [14,15,16,17]. The most frequent of these are complex and condensed tannins that are easily extracted from vegetables, trees and shrubs. The gallic tannins usually occur in gall nuts and lacquer leaves, whereas ellagic tannins are identified in oaks, blackberries and pomegranates. Plant tannins are more abundant in sensitive parts of plants, such as new stems, leaves, and flower buds [18]. The chemical structure and content of tannins vary depending on the species, the stages and growth conditions of the plants (nutrients, light, temperature, and different abiotic and biotic stresses), as well as their biological functions, which is why the sources of extraction also differ considerably [19,20].

*Prunus spinosa* L., known as “blackthorn”, is a member of the *Rosaceae* family, and it is listed as an invasive tree species. Traditionally, the fruits of *P. spinosa* are used for their antiseptic action against inflammation of the oral and pharyngeal mucosa, as an astringent in the treatment of gastrointestinal and respiratory tract diseases, and as a remedy for atherosclerosis [5].

Referring to the flower, it is considered to have protective, anti-inflammatory, diuretic, blood-purifying and spasmolytic activities, as well as vermicidal action, while the branches have shown antihypertensive properties. The leaves of *P. spinosa* have been used for constipation, while its fruits have laxative action [5,21]. Furthermore, its various parts have also been reported to be traditionally used as emmenagogues, diaphoretics, analgesics, antispasmodics, anti-edema and leucorrhoea [22]. Several studies have focused on the in vitro cytotoxic and antioxidant effects of *P. spinosa* fruit extract due to the phytochemicals it contains [5,23,24,25]. Polyphenols found in black cherry, *P. serotina*, seem to enrich this plant’s biochemical defense and suggest that tannins are accumulated mainly in the vacuoles of the upper epidermis and the palisade parenchyma. The main leaf veins display a high density of tannin cells, as observed through anatomical analysis [26]. *P. cerasifera* represents a phytochemical-rich yet underexploited botanical source, with leaves and branches containing significant levels of total phenolics and soluble condensed tannins. Phytochemical analysis revealed that its condensed tannins are predominantly composed of afzelechin/epiafzelechin and catechin/epicatechin subunits, which contribute to the observed antioxidant and tyrosinase inhibitory properties in vitro [27]. The wood of *P. avium* L. contains a high level of condensed tannins and a significant proportion of carbohydrates that are associated with flavonoid structures [28].

Recent research aims to establish that extracts concentration of polyphenols is very effective and protective against chronic degenerative diseases, especially in neoplasms, where the pro-inflammatory context [29] promotes carcinogenesis [30]. However, studies on the chemical composition and biological action of condensed tannins present in the woody part of *Prunus* sp. (branches, bark) are limited or even absent, and this is why we can consider *P. spinosa* L. among the species that have not been scientifically explored enough in terms of the use of its woody parts in new modern therapeutics. In this context, the woody branches of *P. spinosa* represent a particularly interesting bioresource compared with other condensed tannin sources such as leaves, fruits, or bark from related species or other polyphenol-rich plants. Unlike these soft tissues, branches contain a lignocellulosic matrix that may influence both the yield and molecular characteristics of condensed tannins. Investigating this matrix expands current knowledge on alternative, sustainable sources of catechin-type tannins, aligning with circular bioeconomy strategies and the valorization of woody plant residues.

Although *P. spinosa* fruits are widely used in traditional medicine, the woody parts of the plant—especially the branches—have received little attention as potential sources of bioactive constituents. Only a limited number of studies have investigated the phytochemical composition and biological effects of condensed tannins derived from *P. spinosa* branches [31]. This study addresses this knowledge gap by providing a comprehensive chemical and biological characterization of a crude extract derived from *P. spinosa* branches using a green microwave-assisted extraction (MAE) method. The research aims to (i) identify and quantify condensed tannins using HPTLC, (ii) assess antioxidant capacity through HPTLC-DPPH, DPPH and ABTS assays, and (iii) evaluate the extract’s cytotoxic and genotoxic potential using MTT and Comet assays on both normal (Vero) and cancer (HeLa) cell lines. To the best of our knowledge, this is the first study to report the chemical profile and in vitro antioxidant, cytotoxic, and genotoxic effects of condensed tannins extracted from *P. spinosa* L. branches. While the fruits and flowers of this species have been studied previously, the woody biomass remains scientifically underexplored despite its promising phytochemical potential.

## 2. Materials and Methods

### 2.1. The Biomass

The plant material (*P. spinosa* L.) was collected from the Siret Valley region, in Letea Veche (Bacău County, Romania), at the following coordinates: 46.4872° N, 26.9679° E. The botanical identity of *P. spinosa* L. was established based on macroscopic features and verified with reference to specialized botanical literature and herbarium specimens. A formal confirmation of the plant material was provided by biologists from the National Institute of Research and Development for Biological Sciences—Stejarul Research Centre for Biological Sciences, Piatra Neamt, county, Romania. A voucher specimen no. CCBS_00035 was deposited in the institutional collection for reference. The biomass was dried in a well-ventilated room, and the weight loss was measured with an infrared Kern MLS Thermobalance. The raw material was powdered using a Microtom MB550 laboratory mill (Thermo Fisher Scientific, Waltham, MA, USA) to particles with a 0.1 mm medium size and stored in a desiccator. The crude extract was obtained following an unconventional extraction method, respectively, a microwave-assisted extraction using an Ethos X laboratory equipment, Milestone, Italy. Ethanol solution (70% *v*/*v* ethanol) was used as the extraction solvent, considering the high solubility of targeted compounds as well as the cost and use effectiveness. The extraction parameters were 1/10 solid to liquid ratio, 5 min as extraction time, and a 600 W microwave irradiation power. Microwave-assisted extraction (MAE) conditions were defined based on a previous factorial design and response surface modeling study, which optimized the extraction of condensed tannins from *P. spinosa* L. branches. Detailed methodology and statistical validation are available in our previous publication [32]. The crude extract (PS) was separated through filtration and stored at 4 °C prior to phytochemical analysis and biological assays.

### 2.2. Chemicals and Reagents

Reagents of analytical purity were used in experimental research: methanol (99.98%, Chimreactiv, Bucharest, Romania), methanol (99.98%, Chempur, Poland), ethanol (≥96% *v*/*v*) (Chimreactiv, Romania), ethyl acetate (Chromasolv for HPLC, 99.7%; Sigma-Aldrich, St. Louis, MO, USA), toluene (Chromasolv, for HPLC, 99.9%, Sigma Aldrich, USA), formic acid (98–100% for analysis EMSURE^®^ACS, Reag. Ph Eur., Merck KGaA, Darmstadt, Germany), iron (III) chloride (98.5%, extra pure, anhydrous, Roth, Germany), potassium peroxodisulfate (for analysis EMSURE^®^, 99.4%, Merck KGaA, Germany), distilled water, DPPH (2, 2, diphenyl-picryl-hydrazyl) (Sigma-Aldrich, USA); ABTS (2,2′-Azino-bis (3-ethylbenzothiazoline-6-sulfonic acid) (97.5%, Roche Diagnostics GmbH, Mannheim, Germany), reference substances (+)-catechin (≥99%, Extrasynthese, Genay—France), (−)-catechin gallate (≥98%, Sigma—Aldrich, Saint Louis, MO, USA), (−)-gallocatechin (>99%, Carl Roth GmbH+Co.KG, Arlesheim, Switzerland), (−)-gallocatechin gallate (≥99%, Sigma—Aldrich, Saint Louis, MO, USA), (−)-epicatechin (≥97%, Sigma—Aldrich, Saint Louis, MO, USA), (−)-epicatechin gallate (≥98%, Sigma—Aldrich, Saint Louis, MO, USA), (−)-epigallocatechin (≥98%, Extrasynthese, Genay—France) and (−)-epigallocatechin gallate (≥99%, Extra-synthese, Genay—France).

### 2.3. Characterization of Biomass

The proximate analysis was performed in order to characterize the feedstock: humidity (%), total ash (%), and insoluble ash in HCl 100 g/L. Moisture content was determined using a Kern MLS 150—2A thermobalance Kern MLS 150–2A thermobalance (Kern & Sohn GmbH, Balingen, Germany), with a mass measurement range of 0–183 g and a precision of ±0.1 mg. The determination of ash (total ash, insoluble ash in HCl) was carried out according to Ph. Eur, 7th ed., (chapters 2.4.16 and 2.8.1) [33], using a L1003/720 calcination own (Nabertherm GmbH, Lilienthal, Germany), with a maximum temperature of 1100 °C, an analytical balance Precisa/72877/290-9221/6 (Precisa Gravimetrics AG, Dietikon, Switzerland) with a measurement range of 0–183 g ± 0.1 mg and a Precisterm/0408851 electric water bath, thermo-adjustable, with a temperature range of max. 100 °C.

### 2.4. High Performance Thin Layer Chromatography (HPTLC) Analysis of Condensed Tannins from P. spinosa Crude Extract

(a) Preparation of references

The reference solutions for (+)-catechin, (−)-catechin gallate, (−)-gallocatechin, (−)-gallocatechin gallate, (−)-epicatechin, (−)-epicatechin gallate, (−)-epigallocatechin and (−)-epigallocatechin gallate were performed with methanol in order to obtain a 0.02% concentration.

(b) The HPTLC fingerprinting analysis

The identification of condensed tannins from PS was performed using an adapted validated HPTLC method [34,35]. In brief, PS was first fingerprinted using HPTLC and the resulting data (*Rf* values and color hues) were matched or not with the reference substances. Potential matches were confirmed by spectral overlay analysis.

This study used a CAMAG HPTLC system (Muttenz, Switzerland) consisting of a TLC Reprostar 3 visualizer with a 12-bit digital camera and a 16 mm lens, a Linomat IV semi-automatic sample applicator, an ADC2 automated development chamber, and a TLC scanner. The system was operated using WinCATS Version 1.4.3 software, which controls all chromatographic operations and analyses. In order to perform the condensed tannins identification, PS was subjected to the same HPTLC conditions used to establish the database, using the solvent system: toluene: ethyl acetate: formic acid 12:12:2 (*v*/*v*/*v*), as well as ferric chloride as a visualizing reagent.

Sample application and spot development: The chromatographic separation was performed on pre-coated silica gel 60 F254 HPTLC plates (20 cm × 10 cm), used as the stationary phase. Samples were applied as 8 mm bands on the HPTLC plates using a Linomat IV automatic applicator equipped with a 100 µL Hamilton syringe, at a dosage speed of 5 s per microliter. The plate was developed in the solvent system to a distance of 8 cm at room temperature and 45% relative humidity in an Automatic Developing Chamber 2 previously saturated with mobile phase vapors for 20 min.

Photo documentation: The developed plate was air-dried for 10 min. to ensure complete solvent evaporation. A capture of an image at UV 254 nm was performed in order to highlight the separated compounds.

Scanning: Scanning was carried out under white light using the CAMAG Scanner 3 system. Scan settings: slit dimension 6 × 0.3 mm, scanning speed 20 mm/s, data resolution 100 µm/step. The scan mode was single wavelength. The *Rf* table, peak display, spectra and density-grams were registered.

Derivatization: Visualization was achieved by spraying the developed plate with 1% ferric chloride in ethanol, followed by drying at 100 °C for 10 min. The plate was photo-documented in white light.

### 2.5. In Vitro Evaluation of the Antioxidant Activity of P. spinosa Crude Extract

(a) HPTLC–DPPH bioautographic assay

The HPTLC-DPPH bioautographic assay is an effect-directed chromatographic technique commonly used to detect antioxidant constituents in complex mixtures. The HPTLC-DPPH assay was performed according to the protocol described by Cimpoiu [36], with adjustments in solvent composition and detection conditions. The method relies on the decolorization of the stable purple DPPH radical (2,2-diphenyl-1-picrylhydrazyl) by redox-active phytochemicals, including those present in the *P. spinosa* extract. Chromatographic procedures were carried out using a CAMAG HPTLC system.

Samples were applied on silica gel 60 F_254_ plates (10 × 10 cm, Merck) in volumes of 10 µL and 14 µL using a Camag Linomat IV applicator. Bands were 14 mm long and spaced at 12 mm, applied at a rate of 8 s/µL. Plates were pre-washed with methanol and activated at 120 °C for 20 min before application. Development was performed in a saturated vertical chamber at room temperature (20–22 °C) for 30 min using a mobile phase composed of toluene:ethyl acetate:formic acid (12.5:10:1.25, *v*/*v*/*v*). The development distance was 7 cm. After air-drying, plates were sprayed with 0.2% DPPH in methanol and heated at 100 °C for 5 min. Plates were visualized under visible light using a CAMAG Reprostar 3 imaging system. Antioxidant activity was indicated by the appearance of yellow zones against a purple background, corresponding to compounds with free radical scavenging capacity. Chromatograms were analyzed immediately after derivatization and after 24 h to assess the stability of the antioxidant response.

(b) DPPH assay

The antioxidant activity was also traditionally determined as the ability of the extract to scavenge free radicals through 2,2-diphenyl-1-picrylhydrazyl (DPPH) assay [37,38,39]. In brief, 1 mM DPPH solution was prepared as a stock and stored at −20 °C until further use. A series of diluted solutions of PS and standard catechin, with catechin concentrations ranging from 1.227 to 19.643 mg/mL, was prepared. For % (*w*/*v*) inhibition activity, 0.5 mL of each dilute solution was placed in a test tube, and 3.5 mL of DPPH solution was added. Absorbance (A_test_) was recorded at 517 nm after 30 min. of dark incubation. The absorbance of the blank (3.5 mL DPPH + 0.5 mL methanol) (A_blank_) was also measured. The % inhibition activity was calculated using Equation (1):

% Inhibition = (A_blank_ − A_test_)/A_blank_ × 100 (1)

(c) ABTS assay

The ability of PS to neutralize 2,2-azino-bis (3-ethylbenzthiazoline-6-sulfonic acid) (ABTS) free radicals was assessed following a protocol previously described [40]. The absorbance of the ABTS radical solution was measured at 734 nm after 0, 5 and 10 min. of reaction with the extract or catechin standard.

The results for DPPH and ABTS assays were presented as IC_50_ values (*w*/*v*—mg/mL), which refer to the extract concentration that causes a decrease in free radicals of 50%. Catechin was used as a standard antioxidant (control).

### 2.6. In Vitro Evaluation of the Cytotoxic and Genotoxic Effects of P. spinosa Crude Extract

(a) Cell culture and treatment

African green monkey kidney (Vero) cell line (ATCC^®^ CCL-81^™^) and human cervix adenocarcinoma (HeLa) cell line (ATCC^®^ CCL-2^™^) were thawed and reactivate by cultivation in T-75 flasks in Dulbecco Minimum Essential Media (DMEM) supplemented with 10% Fetal Bovine Serum and antibiotics (100 μg/mL Streptomycin, 100 IU/mL Penicillin), and placed in an incubator at 37 °C in humidified atmosphere of 5% CO_2_ and 95% air mixture. After optimal confluence of the two cell lines (>80%), the cells were seeded in 96-well tissue-culture plates (7 × 10^3^ cells/well, for HeLa cells, and 8 × 10^3^ cells/well for the Vero cell line, respectively), and allowed to attach for 24 h prior to treatment. The PS was added in concentrations ranging from 0.05 mg/mL to 5.0 mg/mL, for 48 h. Control cells were maintained in the culture medium only.

(b) MTT assay

The cell viability was assessed through an MTT 3-(4,5-dimethylthiazol-2,5-diphenyl tetrazolium bromide) assay [41,42], which is considered a sensitive method, suitable for adherent cell cultures, based on the ability of living cells to reduce the MTT yellow dye into dark purple formazan crystals by mitochondrial dehydrogenases [43]. The cell monolayer was washed with phosphate-buffered saline (PBS) solution, and a fresh growth medium (100 μL/well) was added, followed by the addition of 10 μL/well MTT (5 mg/mL). The well-plates were placed in an incubator for 3 h at 37 °C under 5% CO_2_ and 95% air conditioning. The reaction was stopped by adding dimethyl sulfoxide (100 μL/well), and the plates were gently shaken to dissolve the insoluble formazan generated after tetrazolium bromide reduction [44,45,46]. The absorbance was spectrophotometrically quantified at 570 nm using a microplate reader (Biochrom, Berlin, Germany).

The percentage of cell viability was calculated using Equation (2):

Cell viability (%) = Sample Abs./Control Abs. × 100(2)

The anticancer effect was expressed as an IC_50_ value which represents 50% inhibition of the cell viability/growth using polynomial graph plots by modeling the percentage of cytotoxicity versus extract concentration.

(c) Comet assay

The alkaline comet assay was used in order to evaluate the genotoxic effect of PS on Vero and HeLa cell lines, considering IC_50_ values. To achieve a positive control, the cells were exposed to a UV lamp (254 nm) for 15 min. For each variant, 3.3 × 10^4^ cells/well (Vero cell line) and 3.0 × 10^4^ cells/well (HeLa cell line) were placed in 24 sterile well plates at 37 °C under a humidified 5% CO_2_ atmosphere to form the cell monolayer. After incubation, the cells were treated with PS for 6 h, and the liquid phases were collected into 15 mL sterile Corning tubes. Each well was washed twice with PBS, followed by trypsin-EDTA solution and centrifugation (1800 rpm, 2 min.). The supernatant was discarded, and the cell pellet was resuspended in 100 μL cold PBS and mixed with preheated low-melting-point agarose (LMA). The cell/agarose mixture was rapidly spread onto the agarose-covered surface of precoated slides (by dipping the slides into molten 1% normal melting agarose (NMA) in PBS) avoid producing bubbles [47]. After gelation (about 5 min.), the slides were immersed in a covered recipient containing cooled lysis buffer and placed at 4 °C in the dark. For electrophoresis, the slides were washed three times (for 20 min) with electrophoresis buffer (0.03 M NaOH, 2 mM Na_2_EDTA, pH ~12.3) and immersed in the tank for 20 min to allow the DNA strands to unwind. The electrophoresis process was conducted for 25 min at 0.6 V/cm, 300 mA, and then the slides were removed and rinsed three times for 5 min. with distilled water. For staining, 50 μL of ethidium bromide 20 μg/mL was spread onto the slide’s surface and incubated for 20 min. The excess stain was removed by rinsing the slides three times for 5 min. of distilled water. The photomicrographs were captured using a Nikon Eclipse 600 epifluorescence microscope equipped with a Nikon Cool Pix 950 digital camera, while the comet analysis was carried out using an OpenComet plugin. The evaluation was performed by calculation of DNA percentage in the head and tail of the comet [48,49].

### 2.7. Data Analysis

DPPH and ABTS antioxidant activities of *P. spinosa* crude extract were performed in triplicate. Results are presented as mean ± standard deviation (SD). IC_50_ values for antioxidant assays were calculated using linear regression analysis. For cytotoxicity assessment (MTT assay), experiments were also conducted in triplicate, and results are shown as mean ± standard error (SE); IC_50_ values were obtained through polynomial regression. The correlation coefficient and regression analysis were performed using Microsoft Excel LTSC Professional Plus 2021. For the comet assay, data were analyzed using Student’s *t* test, with *p* < 0.001 considered statistically significant.

## 3. Results

### 3.1. Characterization of Biomass

The biomass presented a humidity of 8.35 ± 0.05%, which is suitable for the extraction process, a 13% being the maximum for an efficient extraction [33]. The total ash was 1.46 ± 0.09%, while insoluble ash in HCl was 0.02 ± 0.00%.

### 3.2. Characterization of P. spinosa Crude Extract

Condensed tannins identification and quantification—High Performance Thin Layer Chromatography (HPTLC) Analysis

The extract used in HPTLC profiling and the biological activity evaluation in the present study was obtained under optimized microwave-assisted extraction conditions, as previously described by [32]. This extract had a catechin concentration of 3.4 mg/g dry biomass, determined by densitometric analysis.

HPTLC analysis was carried out for PS in order to highlight its condensed tannins compositional profile.

The main features of HPTLC fingerprint are summarized in Table 1, which shows the *Rf* values of the major bands and the assigned compounds. The qualitative phytochemical study carried out by HPTLC highlighted that PS has a varied content of condensed tannins, especially catechins.

**Figure 1:**
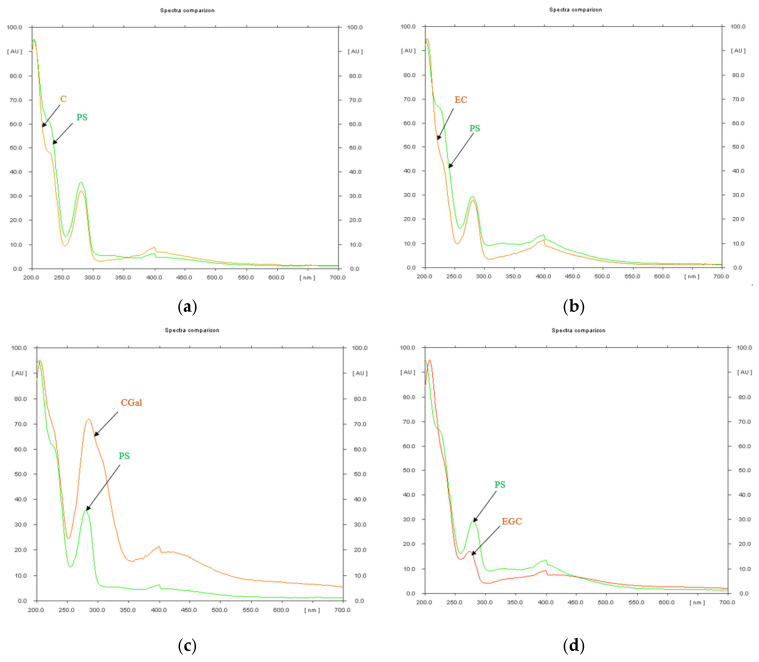

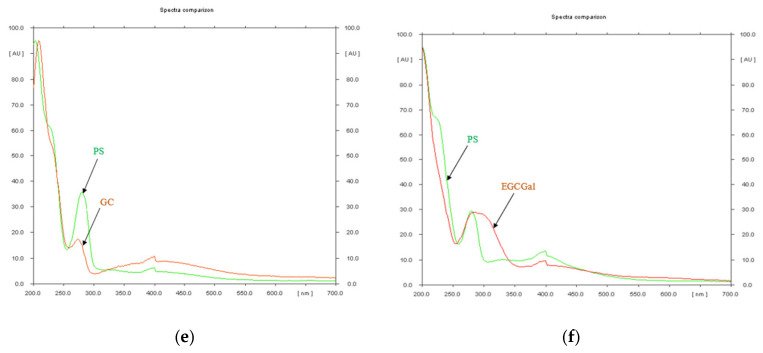
Spectral comparison of the condensed tannins references assigned in *P. spinosa* crude extract (PS)—(**a**) Catechin (C) and PS (**b**) Epicatechin (EC) and PS (**c**) Catechin gallate (CGal)and PS (**d**) Epigallocatechin (EGC) and PS (**e**) Gallocatechin (GC) and PS (**f**) Epigallocatechin gallate (EGCGal) and PS—Screening at 254 nm.

The screening at 254 nm, prior to derivatization, was performed for the quantitative evaluation of the phytochemicals. The references: catechin, epicatechin, catechin gallate, gallocatechin, epigallocatechin and epigallocatechin gallate were assigned in PS, their spectra having the same appearance as the sample (Figure 1), while gallocatechin gallate and epicatechin gallate were not assigned in the investigated extract.

### 3.3. Antioxidant Activity of P. spinosa Crude Extract

(a) HPTLC-DPPH bioautographic assay

**Figure 2:**
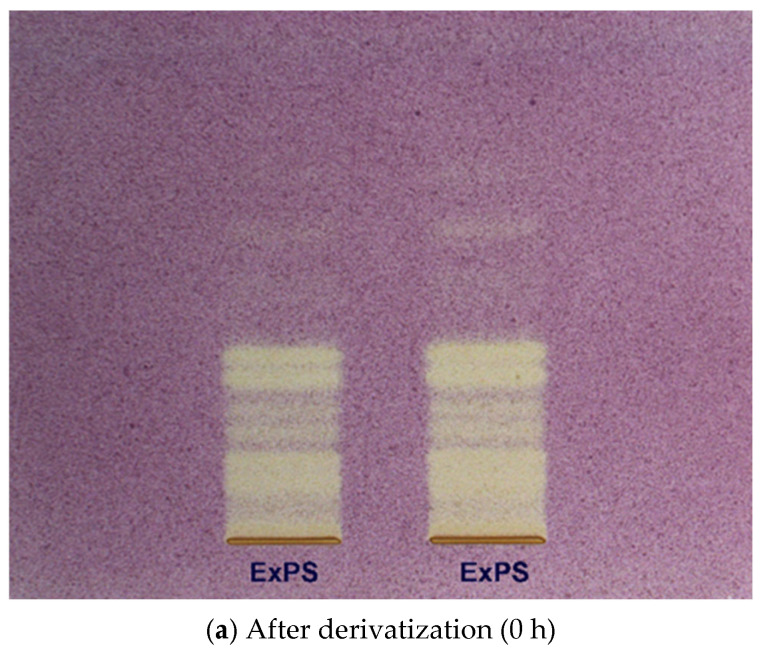

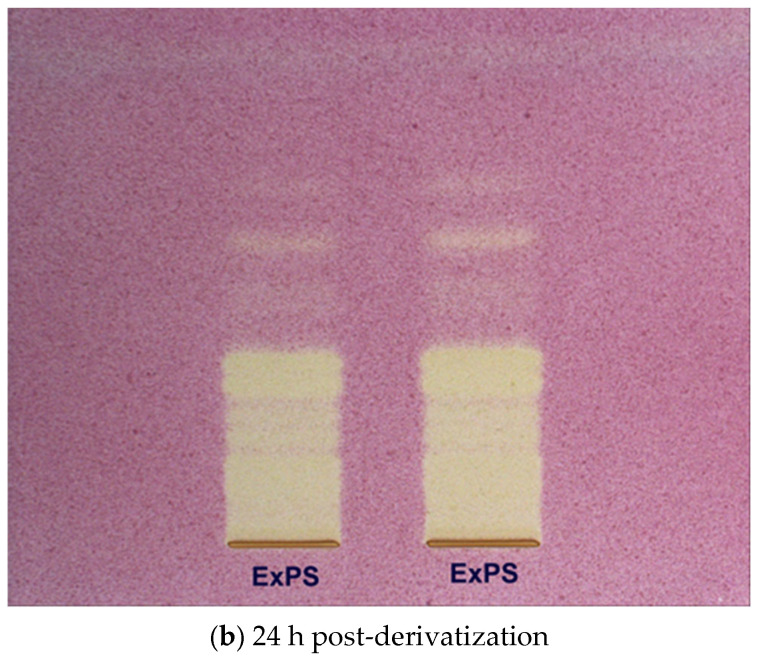
HPTLC plates derivatized with DPPH reagent for *P. spinosa* extract (ExPS) as visualized under visible light after derivatization (0 h)—(**a**) and after 24 h post-derivatization—(**b**).

The antioxidant activity of the *P. spinosa* extract (ExPS) was evaluated using High Performance Thin-Layer Chromatography (HPTLC), revealing that the extract contains a diverse range of catechin-type tannins capable of decolorizing the bands corresponding to antioxidant compounds upon spraying with the DPPH reagent. The HPTLC image (Figure 2) showed the separation of constituents present in the analyzed extract samples, with yellow bands of varying intensities observed—an indication of antioxidant potential. Based on correlation with known phytochemicals identified in the extract, yellow bands at corresponding *Rf* values confirmed the presence of several antioxidant compounds: catechin (*Rf* 0.43), epicatechin (*Rf* 0.40–0.41), epigallocatechin (*Rf* 0.26–0.27), and epigallocatechin gallate (*Rf* 0.22).

(b) DPPH assay

The investigation on the main tannins in *P. spinosa* extract supports further research in revealing the relations between catechin content and total antioxidant activity.

The DPPH radical scavenging activity of PS extract and catechin standard exhibited clear concentration-dependent behavior. For the PS extract, inhibition values ranged from 7.91 ± 0.37 and 89.57 ± 0.11% for the tested concentration range (1.227–19.643 mg/mL), with a strong linear correlation between catechin content and antioxidant activity (R^2^ = 0.9932; regression equation: y = 43.595x + 5.8433). Catechin, used as a reference standard, followed a similar trend, yielding a regression equation of y = 36.542x + 22.004 and a correlation coefficient of R^2^ = 0.9101. Although the catechin regression curve exhibited slightly lower linearity, likely due to the limited concentration range or elevated baseline inhibition, both models consistently demonstrated strong free radical scavenging efficiency. The calculated IC_50_ values were 1.02 ± 0.25 mg/mL for PS extract and 0.76 ± 0.0 mg/mL for catechin, indicating comparable antioxidant efficacy and suggesting possible synergistic interactions among the phenolic constituents in the extract (Table 2).

(c) ABTS Assay

The scavenging activity of the PS and catechin standard against the ABTS^+^ radical cation was assessed at 0, 5, and 10 min. of reaction. At the lowest tested content of catechin (1.227 mg/mL), PS exhibited inhibition values of 10.24 ± 0.28%, 11.73 ± 1.69%, and 11.71 ± 1.58%, respectively. In contrast, catechin standard showed higher inhibition at the same concentration: 65.81 ± 0.60%, 78.25 ± 0.66%, and 82.13 ± 0.81%. At the highest concentration tested (19.643 mg/mL), the PS reached inhibition levels of 89.38 ± 0.19% to 92.10 ± 0.44%, while catechin achieved 94.90 ± 0.24% to 97.12 ± 0.38%, confirming a dose-dependent antioxidant response for both samples. Linear regression models were applied to describe the inhibition-concentration dependence. For the PS, the regression equations and corresponding R^2^ values were: y = 40.509x + 17.187 (R^2^ = 0.8897)—0 min, y = 40.776x + 8.4616 (R^2^ = 0.9745)—5 min, y = 41.519x + 11.586 (R^2^ = 0.9771)—10 min. For catechin standard, the inhibition increased linearly according to the following models: y = 25.448x + 48.925 (R^2^ = 0.9575) at 0 min, y = 25.068x + 49.429 (R^2^ = 0.9255) at 5 min, and y = 27.063x + 46.685 (R^2^ = 0.9345) at 10 min.

The IC_50_ values derived from these regressions confirmed the superior activity of catechin over PS, yet the extract still demonstrated strong antioxidant potential, particularly at longer incubation times. The linearity of the models and the progressive increase in inhibition further support the reliability of the assay and the potential of PS as a multifunctional phytocomplex. Catechin was more active at a lower concentration regardless of the reaction time. Moreover, the action of PS and catechin against the ABTS cation radical decreases with the increase in the reaction time (Table 3). A limitation of this study is the restricted dynamic range for catechin in the ABTS assay, where initial values approached the 50% inhibition threshold. Although IC_50_ could still be derived via polynomial regression, additional concentrations below this range would strengthen curve accuracy.

### 3.4. Assessment of Cytotoxic Effects of P. spinosa Crude Extract

The in vitro screening of the cytotoxic impact of PS was investigated using a normal cell line (Vero) and a tumor line (HeLa), and the cell reactivity to PS action was performed using the MTT assay. In this study, the PS concentration ranged from 0.05 to 5 mg/mL, while the exposure time of cells was 48 h.

**Figure 3:**
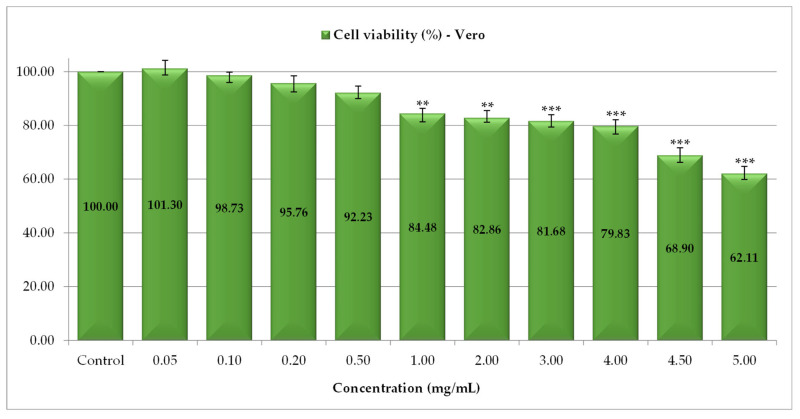
The cytotoxic activity of *P. spinosa* catechin extract against Vero cell line (** *p* < 0.01, *** *p* < 0.001).

For normal Vero cells, low doses (0.05–0.5 mg/mL) of PS slightly interfered with cell development, so the cell viability did not change compared to the control, the percentage of live cells being 92.23% (0.5 mg/mL), respectively, 95.76% (0.2 mg/mL). A decrease in cell viability was reported between 1 and 4 mg/mL of PS and ranged from 84.48% (1 mg/mL) to 79.83% (4 mg/mL), while a second significant reduction in Vero cells proliferation was highlighted using high doses of PS (4.5 and 5 mg/mL), when cells viability was 68.90% and 62.11%, respectively (Figure 3). The results obtained are consistent with those in the literature, which mentioned that catechin structures, in addition to antioxidant action, also exert cytotoxic effects [50].

**Figure 4:**
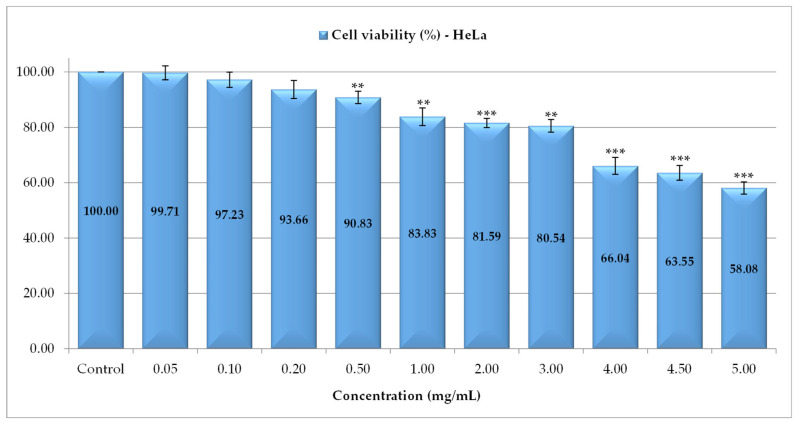
The cytotoxic activity of *P. spinosa* catechin extract against HeLa cell line (** *p* < 0.01, *** *p* < 0.001).

For the HeLa tumor cell line, the cell viability after exposure to PS was also expressed as a percentage relative to the negative control (untreated) (Figure 4). The tests showed three steps of viability decrease: an insignificant decrease at concentrations between 0.05 and 0.5 mg/mL, a second level of decrease occurred for 1, 2 and 3 mg/mL with cell viability between 83.83% (1 mg/mL) and 80.54% (3 mg/mL) and a high rate of inhibition reported at high concentrations (4–5 mg/mL) when the cells viability was between 66.04% (4 mg/mL) and 58.08% (5 mg/mL).

The in vitro significant antitumor impact of PS, evaluated by inhibition of cell development, was generated by the complementarity of two properties that characterize biologically active agents: the cytostatic one due to the negative interference with cellular physiology, and the cytotoxic one due to the decrease in cell viability.

**Figure 5:**
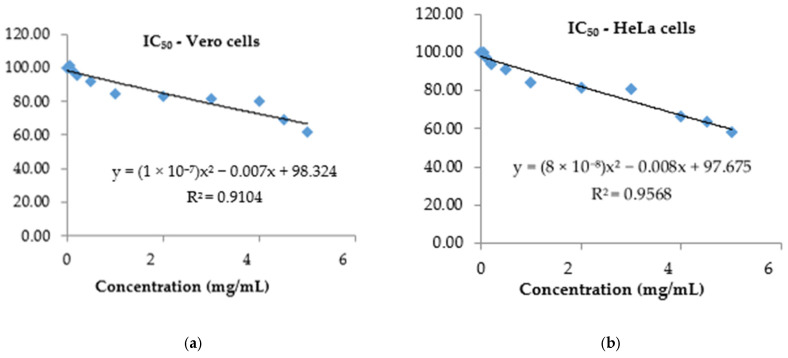
IC_50_ calculated after exposure of Vero (**a**)/HeLa (**b**) cell cultures to *P. spinosa* crude extract.

The correspondence between in vitro development of cells treated with different PS concentrations or not treated allows the assessment of biocompatibility and leads to the calculation of the toxicity index IC_50_—representing the extract concentration responsible for 50% inhibition of viable cells [51]. In the performed tests, a high degree of tolerability of PS at high doses (3–5 mg/mL) was found, proving a reduced toxicity to healthy cells (Figure 5a), with an IC_50_ value of 7.76 mg/mL. The sensitivity of HeLa tumor cells was higher proven by an IC_50_ value of 6.36 mg/mL (Figure 5b). This aspect is a positive one, considering that in antitumor treatments, a low cytotoxic action of the therapeutic agent on normal cells is crucial.

### 3.5. Assessment of Genotoxic Effects of P. spinosa Crude Extract

In order to exhibit the genotoxic effects of PS, interactions with genetic materials were performed, and the DNA fragmentation degree was assessed using the comet test. The comet assay allows for the quantification of cells with different rates of DNA damage [52] by determining the average intensity of the tail, representing the percentage of fragmented DNA detached from the nucleus and migrating to the anode during electrophoresis. There is a linear relationship between the percentage of DNA in the tail and the frequency of DNA breakage [53,54,55,56].

Based on fluorescence microphotographs, the percentage of DNA from the head (Head DNA) and the tail (Tail DNA) of the comet was analyzed. Data were summarized in Table 4.

For normal Vero cell culture, the percentage of DNA at the comet head was 86.59%, while this parameter decreased for the cells treated with PS (IC_50_ was 75.75%). The genotoxic impact of PS on HeLa cells was more intense proved by the size of the tail (almost double compared to control). For both cell lines, the response of cells to the PS is statistically supported, *p* < 0.001. As well, a control (a UV treatment) was used, and in this case, the percentage of comet head was 79.45% for Vero cells and 72.53% for HeLa cells.

**Figure 6:**
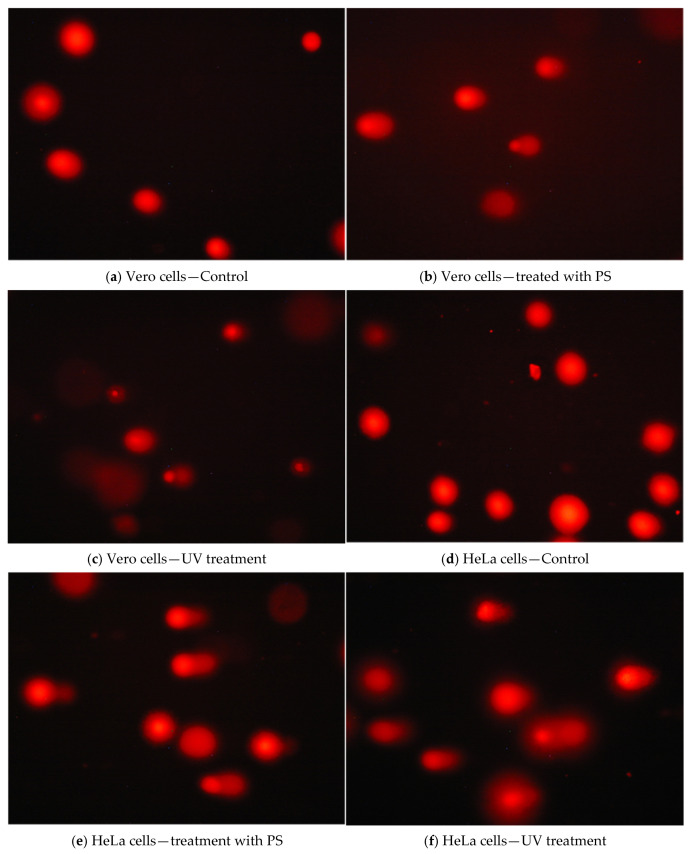
Photomicrographs of stained DNA in a Comet assay for Vero and HeLa cells.

The differential reduction in DNA indicates a potential selectivity effect of the PS. Figure 6 shows the appearance of the nuclei in the control and treated cells for both cell lines.

The results regarding the cyto-physiological behavior of Vero and HeLa cells cultured to the action of *P. spinosa* crude extract reveal accentuated interference in cell proliferation as well as in the function of nuclear genetic material through a significant impact on cell viability and integrity of the DNA.

## 4. Discussions

The present study represents the first comprehensive evaluation of a crude extract derived from *P. spinosa* L. branches, a waste biomass. By integrating phytochemical fingerprinting with in vitro antioxidant, cytotoxic, and genotoxic assessments, this work provides novel insights into the therapeutic potential of woody plant matrices. While antioxidant assays such as HPTLC-DPPH, DPPH and ABTS are well established for condensed tannins, their inclusion here aims to establish a direct correlation between the catechin-rich fingerprint of the branches extract and its functional redox behavior. This link is essential to validate the extract’s bioactivity profile and to support its observed cytotoxic and genotoxic potential. Therefore, the antioxidant assessment serves not merely as a standard screening but as a functional confirmation of the redox-active condensed tannins identified in the extract. The selection of *P. spinosa* branches as a source of condensed tannins is justified by both their chemical composition and their ecological relevance. Unlike the fruits and flowers, which are traditionally used in food or medicinal preparations, the branches represent an abundant and renewable byproduct of pruning and habitat management. From a chemical perspective, the woody tissues exhibit a distinctive polyphenolic fingerprint dominated by catechin and gallocatechin derivatives, comparable or even superior to those reported in other Prunus species. The lignocellulosic matrix of the branches provides a stable environment for phenolic polymerization and may contribute to the formation of complex condensed tannins with enhanced radical-scavenging and metal-chelating capacity. Thus, valorizing *P. spinosa* branches not only provides an alternative to conventional soft-tissue sources but also supports sustainable resource use and circular bioeconomy principles by transforming woody residues into high-value bioactive materials.

The proximate characterization of *P. spinosa* L. branches’ biomass indicated a moisture content that falls within the optimal range for solid–liquid extraction processes, where moisture levels below 13% are considered critical to ensure efficient mass transfer and extract stability [33]. The total ash content and acid-insoluble ash fraction are in accordance with literature reports for lignocellulosic plant materials [57,58], indicating low mineral contamination and confirming the suitability of the biomass for phytochemical applications. The chemical accessibility of phenolics within a lignocellulosic content dominated by cellulose, hemicellulose, and lignin [59] further supports the valorization of *P. spinosa* branches as a viable, underexploited, valuable source of biologically active molecules for nutraceutical or pharmaceutical applications. These findings also support the valorization of *P. spinosa* woody residues in line with current trends of sustainable resource use and circular bioeconomy [60,61,62,63,64].

HPTLC analysis confirmed the presence of flavan-3-ol compounds in the PS, including catechins and their gallate derivatives. These compounds are widely associated with strong antioxidant and bioactive properties. The concentrations of catechin and epicatechin suggest a balanced profile of isomeric flavanols, which may contribute synergistically to the observed biological effects. The presence of gallate derivatives, known for their increased hydrogen-donating and metal-chelating capacity [65,66], further enhances the pharmacological relevance of PS. Compared to other well-studied sources of catechins, such as green tea [67] or cocoa [68], the high concentrations found in *P. spinosa* branches are particularly notable given the woody nature of this biomass. These findings support the efficient extraction of polyphenolic biomarkers by microwave-assisted extraction and indicate that *P. spinosa* branches may serve as a viable alternative source of bioactive flavanols in phytopharmaceutical development. The HPTLC analysis confirmed that *P. spinosa* branches represent a valuable source of condensed tannins (3.4 mg/g dry biomass), in concentrations comparable to or even exceeding those reported in other *Prunus* species (*P. cerasifera*, *P. avium*) [27,69]. Considering that such woody materials are typically regarded as waste, the present findings highlight the potential of *P. spinosa* branches as a novel renewable raw material for the recovery of bioactive flavan-3-ols. Also, catechin levels were quantified, ranging from 1.51 to 8.51 mg/g dw in pruning wood extracts of *P. domestica*, depending on the cultivar [70]. There were reported seasonal variations in catechin content in *P. armeniaca* leaves, with values ranging from 0.50 to 1.5 mg/g dw, increasing toward late summer [71]. In *P. domestica* fruits, catechin was detected at 0.7 mg/g dw, using RP-HPLC [72]. These data validate *P. spinosa* branches as a valuable source of catechins, with potential to serve as a bioactive phytocomplex in nutraceutical or pharmacological applications. These findings are especially relevant given that most phytochemical studies focused on fruits or flowers, while the woody biomass was largely overlooked. The presence of catechins and gallocatechin derivatives increases the chemical complexity of the extract and may contribute to its observed biological activities. This chemical fingerprinting represents a starting point for further pharmacological evaluations and supports the valorization of an underused plant matrix. The chemical characterization of PS highlighted valuable results regarding its phytochemical composition, which fills the gap of knowledge found in the literature.

The antioxidant capacity of the PS was assessed using HPTLC-DPPH, DPPH and ABTS radical scavenging assays, which revealed important antioxidant activity. A large number of HPTLC/HPLC techniques have been developed and successfully applied for the qualitative and quantitative analysis of antioxidants [73,74,75], in which the stable free radical 2,2-diphenyl-1-picrylhydrazyl (DPPH) has often been used as a derivatization reagent for this purpose [76]. Figure 2 shows a distinctly intense yellow band at *Rf* 0.22, significantly more prominent than those observed at other *Rf* values, where the signal intensity is notably lower. The increased coloration over time suggests that the interaction between antioxidant compounds and the DPPH reagent may continue beyond the initial derivatization, enhancing the visibility of active constituents. A comparison between the plates at 0 h and after 24 h reveals a marked intensification of yellow hues, which may reflect a progressive radical-scavenging response or delayed chromogenic reaction. Furthermore, several well-defined yellow bands within the *Rf* range of 0.01–0.22 suggest the presence of additional antioxidant compounds in the *P. spinosa* extract that remain to be identified. This chromatographic behavior is in close agreement with previous reports employing HPTLC–DPPH assays for phytochemical screening of antioxidant constituents in plant matrices. Specifically, Agatonovic-Kustrin and Morton [77] reported yellow decolorized zones on a purple DPPH background corresponding to antioxidant compounds in various plant extracts, validating the selectivity of this visualization method for radical scavengers. Further corroboration arises from Ansari and Dabhi [78], who detected a prominent antioxidant zone at *Rf* = 0.67 in *Abrus precatorius* via HPTLC–DPPH bioautography, confirming the presence of a novel flavonoid with strong radical-scavenging potency. Litewski et al. [79] demonstrated that ethanolic extracts of *Ligustrum vulgare* yield multiple yellow inhibition zones on HPTLC–DPPH plates, primarily attributable to iridoid and flavonoid constituents. Comparable chromatographic responses—yellow bands against a purple background—have also been documented for isoflavone-rich extracts of *Genista saharae* [80] and bark extracts of *Lannea coromandelica* [81], both highlighting phenolic and flavonoid derivatives as key contributors to the observed radical-scavenging zones. Therefore, the observed HPTLC–DPPH plate of *P. spinosa* branches (yellow inhibition band) is consistent with previously reported bioautographic signatures of polyphenol-rich plant matrices. The slight shift in *Rf* value relative to other species can be ascribed to differences in solvent polarity, phytochemical composition, and adsorbent–solute interactions. The antioxidant profile of *P. spinosa* branches is primarily driven by phenolic flavan-3-ols, confirming their key role in the radical-scavenging response observed in the HPTLC–DPPH assay.

The concentrations of the catechin standard used in the DPPH and ABTS spectrophotometric assays were selected to match the range of catechin content estimated in the extract. The IC_50_ value for ABTS radical scavenging activity was calculated using the absorbance recorded at 5 min. PS exhibited inhibition rates of 89.57% and 92.10% against DPPH and ABTS radicals, respectively, with IC_50_ values of 1.02 ± 0.25% and 1.01 ± 0.21%, which confirm a potent antioxidant profile, likely due to the high content of catechins and their gallate and gallocatechin derivatives, as previously identified via HPTLC. Although the IC_50_ values of the extract were higher than those of the pure catechin standard, the extract still exhibited relevant antioxidant activity within a comparable concentration range, likely due to cumulative or synergistic effects among the various phenolic constituents. The results are consistent with the known radical-scavenging abilities of flavan-3-ols and their oligomers, especially in systems with multiple hydroxyl groups and galloyl substitutions, which enhance hydrogen-donating and electron-transfer properties [65,66]. The direct correlation between catechin content and antioxidant performance supports the fact that catechins are major contributors. These results highlight the therapeutic potential of PS, not only as an isolated antioxidant but also as a phytocomplex, potentially offering broader redox modulation compared to single-compound interventions.

The cytotoxic potential of the PS was evaluated using the MTT assay on both HeLa (tumor) and Vero (non-tumor) cell lines. The literature reports few studies on the cytotoxic effect of *Prunus* sp. The extract of *P. spinosa* drupes combined with a nutraceutical activator complex showed antitumor activity in colorectal cancer models, inhibiting the growth and colony formation of HCT116 cells (35%) as compared to the chemotherapy treatment with 5-fluorouracil (80%) [82]. The methanol extract from *P. africana* leaves showed a level of anti-proliferative activity in both breast and colon cancer cells without being toxic to Vero cells [83]. Also, methanolic extracts of *P. africana* (bark) possess high anticancer activities [84]. The PS exhibited a concentration-dependent cytotoxic effect, with a notably higher sensitivity in HeLa cells (IC_50_ = 6.36 mg/mL) compared to Vero cells (IC_50_ = 7.76 mg/mL). This selectivity is an important therapeutic criterion, suggesting that the condensed tannin content might preferentially interfere with tumoral cell metabolism or redox balance. Considering the IC_50_ values, the difference in cytotoxicity between HeLa and Vero cells can be regarded as moderately significant. Similar selective effects were previously described for other *Prunus* extracts enriched in catechins, but this is the first study to demonstrate such effects for *P. spinosa* branch extract, supporting its future evaluation as a potential solution in cancer prevention or therapy. The presence of catechin, epicatechin, and galloylated catechins—previously identified in the extract—likely contributes to this selective effect. Moreover, the lower toxicity observed in Vero cells suggests that the extract does not exert nonspecific cytotoxic effects, supporting its relative safety profile toward non-malignant cells.

The genotoxic potential of the PS was assessed using the Comet assay on both HeLa and Vero cell lines. The Comet assay confirmed the selective genotoxic effect of PS, with HeLa cells showing a markedly higher DNA fragmentation than Vero cells. This selective genotoxicity may be related to oxidative DNA stress or cell-cycle interference mechanisms, particularly relevant in fast-dividing tumor cells. Such behavior mirrors previously reported results for crude extracts from other species but had not been demonstrated until now for *P. spinosa* woody tissues. The genotoxicity profile, when interpreted alongside the cytotoxicity data, suggests a dual mechanism of action—both cytostatic and genotoxic—supporting further mechanistic studies and potential in vivo validation. DNA fragmentation, expressed as tail intensity, was significantly higher in HeLa cells, indicating a selective genotoxic response in tumor cells. In contrast, Vero cells exhibited minimal DNA damage under similar conditions, highlighting the preferential activity of the extract toward malignant phenotypes. This selectivity is of pharmacological interest, as it may reflect mechanisms such as oxidative DNA damage, cell cycle arrest, or interference with topoisomerase activity—mechanisms previously associated with catechin and epigallocatechin gallate derivatives. The observed DNA damage in HeLa cells is consistent with the cytotoxicity data and suggests that PS exerts not only cytostatic but also genotoxic effects on tumor cells, which may enhance its anticancer potential. The findings justify additional studies on the molecular pathways involved and provide a strong rationale for in vivo assessment of PS in antitumor applications.

The determined profile of the *P. spinosa* extract revealed potent antioxidant constituents. Phytochemical evaluation confirmed the dominance of catechic tannins such as catechin, epicatechin, epicatechin gallate, gallocatechin, and proanthocyanidin dimers of type A, a composition consistent with previously reported data for *P. spinosa* branches [85,86]. This catechin-dominated profile aligns closely with other *Rosaceae* species known for high antioxidant capacity, including *Agrimonia eupatoria* and *Potentilla erecta*, both rich in catechin, epicatechin, and procyanidin B3 dimers that contribute to their strong radical-scavenging and antimicrobial effects [87,88]. Likewise, *Geum japonicum* contains O-galloyl-β-glucosides and pedunculagin, phenolic metabolites that exhibit antioxidant and anticoagulant activity [89].

Further comparison within *Rosaceae* demonstrates that *Rosa canina* branches share a similar pattern of polyphenolic and flavonoid constituents associated with immunomodulatory and oxidative-stress-reducing effects [90]. Similar results were reported for *Rosa canina* branch extracts, where extraction kinetics confirmed a high yield of catechic and gallic derivatives [91], as well as for *Hippophae rhamnoides* branches, where high-performance chromatographic analyses indicated the presence of flavonoid and procyanidin complexes with antioxidant properties [92].

Beyond *Rosaceae*, analogous catechin-based antioxidant systems have been described in *Camellia sinensis* (tea leaves), which contain epigallocatechin gallate and related catechins with high radical-scavenging efficiency [93], and in *Diospyros kaki*, whose oligomeric proanthocyanidins exert antiseptic and cardioprotective effects [94]. Comparable condensed-tannin frameworks were also identified in *Machilus pauhoi* [95], *Eucalyptus sideroxylon* [96], and *Picea mariana* [97], where catechin and epicatechin derivatives play a major role in the antioxidant potential of bark and leaf extracts. The chemical similarity between *P. spinosa* and other polyphenol-rich taxa, characterized by catechin, epicatechin, and procyanidin derivatives, suggests a conserved mechanism of redox protection within the *Rosaceae* family. Moreover, analogous condensed tannins have been identified in *Sorbus aucuparia* bark and *Ulmus glabra* bark, species previously investigated for their polyphenolic profiles and antioxidant activity [98,99]. These consistent structural compositions underline the functional homology between *P. spinosa* extract and other antioxidant-rich botanical sources, suggesting that the phenolic matrix of *P. spinosa* branches follows an evolutionarily conserved defense strategy against oxidative stress. These findings demonstrate that the *P. spinosa* branches extract exhibits a phytochemical fingerprint highly analogous to other species across the *Rosaceae* family, confirming its classification among high-potential natural antioxidants with significant pharmacognostic and technological value [32,100,101].

Alongside its antioxidant and cytotoxic properties, *P. spinosa* has also shown promising antimicrobial and antifungal activity in earlier studies using aqueous extracts [102], supporting its potential as a multifunctional phytocomplex. Furthermore, previous research on *P. spinosa* leaf extracts demonstrated significant antioxidant, antidiabetic, antimicrobial, and antitumor activities, largely attributed to their high phenolic and anthocyanin content [103], sustaining the phytopharmacological relevance of this species beyond its traditional applications. The antioxidant and selective cytotoxic activities reported for bark extracts of *Prunus* species such as *P. ceylanica* and *P. padus* [104,105], attributed to their high content of phenolic compounds and condensed tannins, support our findings regarding the cytotoxic and genotoxic potential of the PS, suggesting a similar mechanism of action involving controlled oxidative stress induction in tumor cells. Overall, the exploitation of woody residues from *P. spinosa* for obtaining condensed tannins aligns with current sustainability goals and offers a practical alternative to fruit- or leaf-based extractions, which often compete with food or medicinal uses. These combined biological effects suggest that condensed tannins from *P. spinosa* may exert their activity through multiple mechanisms. Additionally, the use of *P. spinosa* branches contributes to the valorization of a biomass typically regarded as waste, aligning with principles of sustainability and circular bioeconomy.

The present study has some limitations that should be acknowledged. The biological assays were conducted in vitro and used a crude extract, which may contain a complex mixture of bioactive compounds acting synergistically. Therefore, the specific molecular mechanisms underlying the cytotoxic and genotoxic effects of the condensed tannins could not be fully elucidated. Further studies involving fractionation, compound isolation, and in vivo validation are required to confirm these effects and to establish the pharmacological relevance of the extract. Despite these limitations, this work provides important insight into the potential of P. spinosa woody branches as an alternative, sustainable source of condensed tannins. The study demonstrates that branch-derived catechin extract obtained by a green microwave-assisted process can exhibit both strong antioxidant capacity and selective cytotoxicity toward tumor cells. Thus, the findings support the valorization of woody residues in a circular bioeconomy framework and open new perspectives for the development of multifunctional phytochemical ingredients. Further work will include extended biological and pharmacological investigations, such as enzymatic inhibition studies, cellular oxidative stress modulation assays, and in vivo models, to confirm the mechanisms suggested by the current in vitro data.

## 5. Conclusions

This study provides a comprehensive characterization of condensed tannin from crude extract obtained from *P. spinosa* L. branches using a green microwave-assisted extraction (MAE) approach. The extract demonstrated a diverse profile of catechins and their derivatives, as confirmed by HPTLC analysis.

The HPTLC–DPPH bioautographic assay confirmed the presence of potent antioxidant constituents in the *P. spinosa* branches extract, as evidenced by the formation of distinct yellow inhibition zones, indicating a high radical-scavenging capacity primarily attributable to condensed tannins and catechin derivatives.

The extract exhibited potent antioxidant activity in both DPPH and ABTS assays, with strong radical scavenging capacity that correlated with total phenolic content. Moreover, PS showed selective cytotoxic effects, significantly reducing viability in HeLa tumor cells while exerting low toxicity on normal Vero cells. The Comet assay further supported the selective genotoxic potential of PS, with higher DNA damage observed in tumor cells.

These results support the valorization of *P. spinosa* woody biomass—a traditionally underutilized plant matrix—as a promising source of bioactive condensed tannins. The dual antioxidant and antitumor activities revealed by this study provide a strong rationale for further mechanistic investigations and in vivo validation. PS extract may be considered a valuable candidate for the development of natural antioxidant and anticancer formulations.

## Figures and Tables

**Table 1:** *Rf* values from HPTLC fingerprint of *P. spinosa* crude extract.

*Rf* Value	Reference Substance	Assigned Substance in *P. spinosa* Catechin Crude Extract
0.20, 0.23, 0.24		Other tannins without correspondence in the applied standards
0.22	Epigallocatechin gallate	Epigallocatechin gallate
0.26	Gallocatechin gallate	-
0.26–0.27	Epigallocatechin	Epigallocatechin
0.29–0.30	Gallocatechin	Gallocatechin
0.32		Other tannins without correspondence in the applied standards
0.35–0.36	Epicatechin gallate	-
0.38–0.39	Catechin gallate	Catechin gallate
0.40–0.41	Epicatechin	Epicatechin
0.43	Catechin	Catechin

**Table 2:** IC_50_ values obtained for *P. spinosa* crude extract and reference substance catechin against DPPH reagent.

Extract/Control	IC_50_ (mg/mL)
PS	1.02 ± 0.25
Catechin—reference substance	0.76 ± 0.0

**Table 3:** IC_50_ values obtained for PS and reference substance catechin solutions against the radical cation ABTS.

Extract/Control	IC_50_ (mg/mL)		
	0 min	5 min	10 min
PS	0.81 ± 0.10	1.0188 ± 0.21	0.9255 ± 0.19
Catechin	0.023 ± 0.00	0.0424 ± 0.00	0.122 ± 0.04

**Table 4:** Effect of *P. spinosa* crude extract (calculated dose for IC_50_) on DNA integrity in Vero and HeLa cells.

	Vero			HeLa		
	Head DNA% Mean ± SEM	Tail DNA% Mean ± SEM	*p*	Head DNA% Mean ± SEM	Tail DNA% Mean ± SEM	*p*
Control	86.59 ± 1.36	13.41 ± 1.36		81.13 ± 1.19	18.87 ± 1.19	
IC_50_	75.76 ± 1.24	24.76 ± 1.25	<0.001	67.64 ± 1.27	32.36 ± 1.27	<0.001
UV	79.45 ± 1.41	20.55 ± 1.41	<0.001	72.53 ± 1.80	27.47 ± 1.80	<0.001

## Data Availability

The original contributions presented in this study are included in the article. Further inquiries can be directed to the corresponding author.

## References

[1] Georgiev M.I. (2014). Natural Products Utilization. Phytochem. Rev..

[2] Bernardini S., Tiezzi A., Laghezza Masci V., Ovidi E. (2018). Natural Products for Human Health: An Historical Overview of the Drug Discovery Approaches. Nat. Prod. Res..

[3] Mostafa A.A., Al-Askar A.A., Almaary K.S., Dawoud T.M., Sholkamy E.N., Bakri M.M. (2018). Antimicrobial Activity of Some Plant Extracts Against Bacterial Strains Causing Food Poisoning Diseases. Saudi J. Biol. Sci..

[4] Sabatini L., Fraternale D., Di Giacomo B., Mari M., Albertini M.C., Gordillo B., Rocchi M.B.L., Sisti D., Coppari S., Semprucci F. (2020). Chemical Composition, Antioxidant, Antimicrobial and Anti-Inflammatory Activity of *Prunus spinosa* L. Fruit Ethanol Extract. J. Funct. Foods.

[5] Marchelak A., Owczarek A., Matczak M., Pawlak A., Kolodziejczyk-Czepas J., Nowak P., Olszewska M.A. (2017). Bioactivity Potential of *Prunus spinosa* L. Flower Extracts: Phytochemical Profiling, Cellular Safety, Pro-Inflammatory Enzymes Inhibition and Protective Effects Against Oxidative Stress In Vitro. Front. Pharmacol..

[6] Kour A. (2014). Plants Exhibiting Potential for Cancer Treatment. Int. J. Pharm. Sci. Rev. Res..

[7] Takeoka G.R., Dao L.T. (2003). Antioxidant Constituents of Almond [*Prunus dulcis* (Mill.) D.A. Webb] Hulls. J. Agric. Food Chem..

[8] Kumar S., Pandey A.K. (2013). Chemistry and biological activities of flavonoids: An overview. Sci. World J..

[9] Cushnie T.P., Lamb A.J. (2011). Recent advances in understanding the antibacterial properties of flavonoids. Int. J. Antimicrob. Agents.

[10] Daglia M. (2012). Polyphenols as antimicrobial agents. Curr. Opin. Biotechnol..

[11] Chae S., Lee J., Park S. (2013). Recent studies on flavonoids and their antioxidant activities. EXCLI J..

[12] Gourlay G., Constabel C.P. (2019). Condensed Tannins Are Inducible Antioxidants and Protect Hybrid Poplar Against Oxidative Stress. Tree Physiol..

[13] Berard N.C., Wang Y., Wittenberg K.M., Krause D.O., Coulman B.E., McAllister T.A., Ominski K.H. (2011). Condensed Tannin Concentrations Found in Vegetative and Mature Forage Legumes Grown in Western Canada. Can. J. Plant Sci..

[14] Kyamuhangire W., Krekling T., Reed E., Pehrson R. (2006). The Microstructure and Tannin Content of Banana Fruit and Their Likely Influence on Juice Extraction. J. Sci. Food Agric..

[15] Sarneckis C.J., Dambergs R.G., Jones P., Mercurio M., Herderich M.J., Smith P.A. (2006). Quantification of Condensed Tannins by Precipitation with Methyl Cellulose: Development and Validation of an Optimised Tool for Grape and Wine Analysis. Aust. J. Grape Wine Res..

[16] Ortiz J., Marín-Arroyo M.-R., Noriega-Domínguez M.-J., Navarro M., Arozarena I. (2013). Color, Phenolics, and Antioxidant Activity of Blackberry (*Rubus glaucus* Benth.), Blueberry (*Vaccinium floribundum* Kunth.), and Apple Wines from Ecuador: Fruit Wines from Ecuador. J. Food Sci..

[17] Figueroa-Espinoza M.C., Zafimahova A., Alvarado P.G.M., Dubreucq E., Poncet-Legrand C. (2015). Grape Seed and Apple Tannins: Emulsifying and Antioxidant Properties. Food Chem..

[18] Terrill T.H., Rowan A.M., Douglas G.B., Barry T.N. (1992). Determination of Extractable and Bound Condensed Tannin Concentrations in Forage Plants, Protein Concentrate Meals and Cereal Grains. J. Sci. Food Agric..

[19] Jin L., Wang Y., Iwaasa A.D., Xu Z., Schellenberg M.P., Zhang Y.G., Liu X.L., McAllister T.A. (2012). Effect of Condensed Tannins on Ruminal Degradability of Purple Prairie Clover (*Dalea purpurea* Vent.) Harvested at Two Growth Stages. Anim. Feed. Sci. Technol..

[20] Li Y., Iwaasa A.D., Wang Y., Jin L., Han G., Zhao M. (2014). Condensed Tannins Concentration of Selected Prairie Legume Forages as Affected by Phenological Stages During Two Consecutive Growth Seasons in Western Canada. Can. J. Plant Sci..

[21] Karakas N., Okur M.E., Ozturk I., Ayla S., Karadağ A.E., Polat D.Ç. (2019). Antioxidant Activity and Cytotoxic Effects of *Prunus spinosa* L. Fruit Extract on Various Cancer Cell Lines. MMJ.

[22] Vokou D., Katradi K., Kokkini S. (1993). Ethnobotanical Survey of Zagori (Epirus, Greece), a Renowned Centre of Folk Medicine in the Past. J. Ethnopharmacol..

[23] Meschini S., Pellegrini E., Condello M., Occhionero G., Delfine S., Condello G., Mastrodonato F. (2017). Cytotoxic and Apoptotic Activities of *Prunus spinosa* Trigno Ecotype Extract on Human Cancer Cells. Molecules.

[24] Mohammed S.B., Upyr T.V., Shapoval O.M., Lenchyk L.V., Georgiev K. (2019). Determination of phenolic compounds in *Prunus domestica* fruits extract and its pharmacological activity. JofIMAB.

[25] Uysal S. (2020). Comparative Antioxidant Capacity and Enzyme Inhibitory Effect of Extracts from Prunus Avium Leaves. Kastamonu Üniversitesi Orman Fakültesi Derg..

[26] Halarewicz A. (2011). Tissue localization of the condensed tannins in the leaves of the black cherry, *Prunus serotina* ehrh. Electron. J. Pol. Agric. Univ..

[27] Song W., Qin S.-T., Fang F.-X., Gao Z.-J., Liang D.-D., Liu L.-L., Tian H.-T., Yang H.-B. (2018). Isolation and Purification of Condensed Tannin from the Leaves and Branches of *Prunus cerasifera* and Its Structure and Bioactivities. Appl. Biochem. Biotechnol..

[28] Cesprini E., De Iseppi A., Giovando S., Tarabra E., Zanetti M., Šket P., Marangon M., Tondi G. (2022). Chemical Characterization of Cherry (*Prunus avium*) Extract in Comparison with Commercial Mimosa and Chestnut Tannins. Wood Sci. Technol..

[29] Roy S., Trinchieri G. (2017). Microbiota: A Key Orchestrator of Cancer Therapy. Nat. Rev. Cancer.

[30] Cardona F., Andrés-Lacueva C., Tulipani S., Tinahones F.J., Queipo-Ortuño M.I. (2013). Benefits of Polyphenols on Gut Microbiota and Implications in Human Health. J. Nutr. Biochem..

[31] Negrean O.-R., Farcas A.C., Pop O.L., Socaci S.A. (2023). Blackthorn—A Valuable Source of Phenolic Antioxidants with Potential Health Benefits. Molecules.

[32] Ciuperca (Apreutesei) O.T., Ionescu E., Secula M.S., Volf I. (2023). Microwave-Assisted Extraction of Condensed Tannins from Branches of *Prunus spinosa* L.: Response Surface Modeling and Optimization. Processes.

[33] Council of Europe (2012). European Pharmacopoeia.

[34] Wagner H., Bladt S. (2009). Plant Drug Analysis: A Thin Layer Chromatography Atlas.

[35] Reich E., Schibli A. (2007). High-Performance Thin-Layer Chromatography for the Analysis of Medicinal Plants.

[36] Cimpoiu D.C. (2006). Analysis of Some Natural Antioxidants by Thin-Layer Chromatography and High-Performance Thin-Layer Chromatography. J. Liq. Chromatogr. Relat. Technol..

[37] Brand-Williams W., Cuvelier M.E., Berset C. (1995). Use of a Free Radical Method to Evaluate Antioxidant Activity. LWT FoodSci. Technol..

[38] Molyneux P. (2004). The Use of the Stable Free Radical Diphenylpicrylhydrazyl (DPPH) for Estimating Antioxidant. Songklanakarin J. Sci. Technol..

[39] Bujor O.-C., Le Bourvellec C., Volf I., Popa V.I., Dufour C. (2016). Seasonal Variations of the Phenolic Constituents in Bilberry (*Vaccinium myrtillus* L.) Leaves, Stems and Fruits, and Their Antioxidant Activity. Food Chem..

[40] Zheleva-Dimitrova D., Nedialkov P., Kitanov G. (2010). Radical Scavenging and Antioxidant Activities of Methanolic Extracts from *Hypericum* Species Growing in Bulgaria. Phcog. Mag..

[41] Mosmann T. (1983). Rapid Colorimetric Assay for Cellular Growth and Survival: Application to Proliferation and Cytotoxicity Assays. J. Immunol. Methods.

[42] Laville N., Aït-Aïssa S., Gomez E., Casellas C., Porcher J.M. (2004). Effects of Human Pharmaceuticals on Cytotoxicity, EROD Activity and ROS Production in Fish Hepatocytes. Toxicology.

[43] Denizot F., Lang R. (1986). Rapid Colorimetric Assay for Cell Growth and Survival. J. Immunol. Methods.

[44] Berridge M.V., Herst P.M., Tan A.S. (2005). Tetrazolium Dyes as Tools in Cell Biology: New Insights into Their Cellular Reduction. Biotechnology Annual Review.

[45] van Meerloo J., Kaspers G.J.L., Cloos J., Cree I.A. (2011). Cell Sensitivity Assays: The MTT Assay. Cancer Cell Culture.

[46] Stockert J.C., Blázquez-Castro A., Cañete M., Horobin R.W., Villanueva Á. (2012). MTT Assay for Cell Viability: Intracellular Localization of the Formazan Product Is in Lipid Droplets. Acta Histochem..

[47] Olive P.L., Banáth J.P. (2006). The Comet Assay: A Method to Measure DNA Damage in Individual Cells. Nat. Protoc..

[48] Kumaravel T.S., Vilhar B., Faux S.P., Jha A.N. (2009). Comet Assay Measurements: A Perspective. Cell Biol. Toxicol..

[49] Singh N.P., Stephens R.E., Schneider E.L. (1994). Modifications of Alkaline Microgel Electrophoresis for Sensitive Detection of DNA Damage. Int. J. Radiat. Biol..

[50] Murakami C., Hirakawa Y., Inui H., Nakano Y., Yoshida H. (2002). Effect of Tea Catechins on Cellular Lipid Peroxidation and Cytotoxicity in HepG2 Cells. Biosci. Biotechnol. Biochem..

[51] Damiani E., Solorio J.A., Doyle A.P., Wallace H.M. (2019). How Reliable Are In Vitro IC_50_ Values? Values Vary with Cytotoxicity Assays in Human Glioblastoma Cells. Toxicol. Lett..

[52] Serpeloni J.M., Bisarro dos Reis M., Rodrigues J., Campaner dos Santos L., Vilegas W., Varanda E.A., Dokkedal A.L., Colus I.M.S. (2008). In Vivo Assessment of DNA Damage and Protective Effects of Extracts from Miconia Species Using the Comet Assay and Micronucleus Test. Mutagenesis.

[53] Basri D.F., Alamin Z.A.Z., Chan K.M. (2015). Assessment of Cytotoxicity and Genotoxicity of Stem Bark Extracts from *Canarium odontophyllum* Miq. (Dabai) Against HCT 116 Human Colorectal Cancer Cell Line. BMC Complement Altern. Med..

[54] Al-Faifi Z., Masrahi Y., Aly M., Al-Turki T., Dardeer T. (2017). Evaluation of Cytotoxic and Genotoxic Effects of *Euphorbia triaculeata* Forssk. Extract. Asian Pac. J. Cancer Prev..

[55] Kahaliw W., Hellman B., Engidawork E. (2018). Genotoxicity Study of Ethiopian Medicinal Plant Extracts on HepG2 Cells. BMC Complement Altern. Med..

[56] Paul S., Chakraborty S., Mukherjee A., Kundu R. (2015). Evaluation of Cytotoxicity and DNA Damaging Activity of Three Plant Extracts on Cervical Cancer Cell Lines. Int. J. Pharm. Sci. Rev. Res..

[57] Dibdiakova J., Wang L., Li H. (2015). Characterization of Ashes from Pinus Sylvestris Forest Biomass. Energy Procedia.

[58] Protásio T.d.P., Tonoli G.H.D., Guimarães Júnior M., Bufalino L., Couto A.M., Trugilho P.F. (2012). Correlações Canônicas Entre as Características Químicas e Energéticas de Resíduos Lignocelulósicos. CERNE.

[59] Okolie J.A., Nanda S., Dalai A.K., Kozinski J.A. (2021). Chemistry and Specialty Industrial Applications of Lignocellulosic Biomass. Waste Biomass Valor..

[60] Dessie W., Luo X., He F., Liao Y., Duns G., Qin Z. (2023). Lignin valorization: A crucial step towards full utilization of biomass, zero waste and circular bioeconomy. Biocatal. Agric. Biotechnol..

[61] Devi A., Bajar S., Kour H., Kothari R., Pant D., Singh A. (2022). Lignocellulosic Biomass Valorization for Bioethanol Production: Circular Bioeconomy Approach. Bioenerg. Res..

[62] Dhiman S., Kaur P., Narang J., Mukherjee G., Thakur B., Kaur S., Tripathi M. (2024). Fungal bioprocessing for circular bioeconomy: Exploring lignocellulosic waste valorization. Mycology.

[63] Yadav A., Sharma V., Tsai M., Chen C.-W., Sun P.-P., Nargotra P., Wang J.-X., Dong C. (2023). Development of lignocellulosic biorefineries for the sustainable production of biofuels: Towards circular bioeconomy. Bioresour. Technol..

[64] Volf I., Popa V.I. (2018). Integrated Processing of Biomass Resources for Fine Chemical Obtaining. Biomass as Renewable Raw Material to Obtain Bioproducts of High-Tech Value.

[65] Zhong Y., Ma C.M., Shahidi F. (2012). Antioxidant and antiviral activities of lipophilic epigallocatechin gallate (EGCG) derivatives. Funct. Foods.

[66] Oliveira M.R., Nabavi S.F., Daglia M., Rastrelli L., Nabavi S.M. (2016). Epigallocatechin gallate and mitochondria-A story of life and death. Pharmacol. Res..

[67] Kim H.S., Quon M.J., Kim J.A. (2014). New insights into the mechanisms of polyphenols beyond antioxidant properties; lessons from the green tea polyphenol, epigallocatechin 3-gallate. Redox Biol..

[68] Coșarcă S., Tanase C., Muntean D.L. (2019). Therapeutic Aspects of Catechin and Its Derivatives—An Update. Acta Biol. Marisiensis.

[69] Agarwal C., Hofmann T., Vršanská M., Schlosserová N., Visi-Rajczi E., Voběrková S., Pásztory Z. (2021). In vitro antioxidant and antibacterial activities with polyphenolic profiling of wild cherry, the European larch and sweet chestnut tree bark. Eur. Food Res. Technol..

[70] Ortega-Vidal J., Ruiz-Martos L., Salido S., Altarejos J. (2023). Proanthocyanidins in Pruning Wood Extracts of Four European Plum (*Prunus domestica* L.) Cultivars: Quantitative Analysis. Chem. Biodivers..

[71] Uğur Y., Erdoğan S., Yılmaz İ., Başgel S. (2018). Variation of Composition of Phenolic Compounds in the Apricot (*Prunus armeniaca* L.) Leaves by Seasons. J. Nat. Prod. Plant Resour..

[72] Amir M., Mujeeb M., Ahmad S., Akhtar M., Kamal Y.T., Ashraf K. (2013). Simultaneous Quantitative HPLC Analysis of Ascorbic Acid, Gallic Acid, and Catechin in Punica granatum, Tamarindus indica and Prunus domestica. Planta Med..

[73] Jasprica I., Bojić M., Mornar A., Bešić E., Bučan K., Medić-Šarić M. (2007). Evaluation of Antioxidative Activity of Croatian Propolis Samples Using DPPH· and ABTS^+^ Stable Free Radical Assays. Molecules.

[74] Zhao J., Zhang J.S., Yang B., Lv G.P., Li S.P. (2017). Free Radical Scavenging Activity and Characterization of Sesquiterpenoids in Four Species of Curcuma Using a TLC Bioautography Assay and GC–MS Analysis. Molecules.

[75] Wang J., Yue Y.D., Tang F., Sun J. (2012). Screening and Analysis of the Potential Bioactive Components in Rabbit Plasma After Oral Administration of Hot-Water Extracts from Leaves of Bambusa textilis McClure. Molecules.

[76] Kusznierewicz B., Piekarska A., Mrugalska B., Konieczka P., Namieśnik J., Bartoszek A. (2012). Phenolic Composition and Antioxidant Properties of Polish Blue-Berried Honeysuckle Genotypes by HPLC-DAD-MS, HPLC Post Column Derivatization with ABTS or FC, and TLC with DPPH Visualization. J. Agric. Food Chem..

[77] Agatonovic-Kustrin S., Morton D.W. (2018). HPTLC–Bioautographic Methods for Selective Detection of the Antioxidant and α-Amylase Inhibitory Activity in Plant Extracts. MethodsX.

[78] Ansari H.I., Dabhi R.C., Trivedi P.G., Thakar M.S., Maru J.J., Sindhav G.M. (2023). Isolation and Characterization of an Undescribed Flavonoid from *Abrus precatorius* L. Based on HPTLC-DPPH Bioautography and Its Cytotoxicity Evaluation. Future J. Pharm. Sci..

[79] Litewski S., Mróz M., Bartoszek A., Kusznierewicz B. (2023). Post-Chromatographic Derivatization Coupled with Mass Spectrometry as a Method of Profiling and Identification of Antioxidants; *Ligustrum vulgare* Phytocomplex as an Example. Molecules.

[80] Meriane D., Genta-Jouve G., Kaâbeche M., Michel S., Boutefnouchet S. (2014). Rapid Identification of Antioxidant Compounds of Genista saharae Coss. & Dur. by Combination of DPPH Scavenging Assay and HPTLC-MS. Molecules.

[81] Rajesh R. (2021). Phytochemical Screening, HPTLC Finger Print and In Vitro Antioxidant Activity of Bark Extracts of *Lannea coromandelica* (Houtt.) Merr. Indian J. Pharm. Educ. Res..

[82] Condello M., Pellegrini E., Spugnini E.P., Baldi A., Amadio B., Vincenzi B., Occhionero G., Delfine S., Mastrodonato F., Meschini S. (2019). Anticancer Activity of “Trigno M”, Extract of *Prunus spinosa* Drupes, Against In Vitro 3D and In Vivo Colon Cancer Models. Biomed. Pharmacother..

[83] Nabende P., Karanja S., Mwatha J., Wachira S. (2015). Anti-Proliferative Activity of Prunus Africana, Warburgia stuhlmannii and Maytenus senegalensis Extracts in Breast and Colon Cancer Cell Lines. Eur. J. Med. Plants.

[84] Onyancha J.M., Gikonyo N.K., Wachira S.W., Mwitari P.G., Gicheru M.M. (2018). Anticancer Activities and Safety Evaluation of Selected Kenyan Plant Extracts Against Breast Cancer Cell Lines. J. Pharmacogn. Phytother..

[85] Pinacho R., Cavero R.Y., Astiasarán I., Ansorena D., Calvo M.I. (2015). Phenolic Compounds of Blackthorn (*Prunus spinosa* L.) and Influence of In Vitro Digestion on Their Antioxidant Capacity. J. Funct. Foods.

[86] Jang G.H., Kim H.W., Lee M.K., Jeong S.Y., Bak A.R., Lee D.Y., Kim J.B. (2018). Characterization and Quantification of Flavonoid Glycosides in the Prunus Genus by UPLC-DAD-QTOF/MS. Saudi J. Biol. Sci..

[87] Granica S., Krupa K., Kłębowska A., Kiss A.K. (2013). Development and Validation of an HPLC-DAD-CAD-MS^3^ Method for Qualitative and Quantitative Standardization of Polyphenols in *Agrimoniae eupatoriae* herba. J. Pharm. Biomed. Anal..

[88] Tomczyk M., Latté K.P. (2009). Potentilla—A Review of Its Phytochemical and Pharmacological Profile. J. Ethnopharmacol..

[89] Xie Y.W., Xu H.X., Dong H., Fiscus R.R., But P.P.H. (2007). Role of Nitric Oxide in the Vasorelaxant and Hypotensive Effect of Extracts and Purified Tannins from *Geum japonicum*. J. Ethnopharmacol..

[90] Crețu R., Mihăilescu R., Mitroi G., Iacob E., Ionescu E. (2007). Cercetări Privind Obținerea Unor Produse Fitoterapeutice cu Acțiune Antioxidantă din Partea Lemnoasă de *Vitis vinifera* L., *Rosa canina* L., și *Hippophae rhamnoides* L. Obținerea și Caracterizarea Unui Extract Complex cu Acțiune Antioxidantă. Rev. Med. Chir. Soc. Med. Nat. Iași.

[91] Ciupercă O.T., Ţebrencu C.E., Lăzar L., Volf I. (2020). Kinetic Study on Solid–Liquid Extraction of Condensed Tannins from Rosa canina Branches and Stems. Rev. Chim..

[92] Apreutesei O.T., Ţebrencu C.E., Ionescu E. (2024). Exploring the Phytochemical Profile and Quality Control of *Hippophae rhamnoides* (*Sea buckthorn*) Branches. Ann. Acad. Rom. Sci. Phys. Chem. Sci..

[93] Sharangi A.B. (2009). Medicinal and Therapeutic Potentialities of Tea (*Camellia sinensis* L.)—A Review. Food Res. Int..

[94] Zhang Y., Shi P., Qu H. (2011). Antioxidant and Anticancer Properties of Persimmon (*Diospyros kaki*) Condensed Tannins. Food Chem..

[95] Wei S.D., Chen R.Y., Liao M.M., Tu N.W., Zhou H.C., Lin Y.M. (2011). Antioxidant Condensed Tannins from *Machilus pauhoi* Leaves. J. Med. Plants Res..

[96] Miranda I., Lima L., Quilhó T., Knapic S., Pereira H. (2016). The Bark of *Eucalyptus sideroxylon* as a Source of Phenolic Extracts with Antioxidant Properties. Ind. Crops Prod..

[97] Diouf P.N., Stevanovic T., Cloutier A. (2009). Study on Chemical Composition, Antioxidant and Anti-Inflammatory Activities of Hot Water Extract from *Picea mariana* Bark and Its Proanthocyanidin-Rich Fractions. Food Chem..

[98] Ionescu E., Tebrencu C.E., Ciupercă O.T., Vochița G. (2018). HPTLC Investigation of Phenolic Compounds from the Extracts of *Ulmus glabra* Huds. Bark. An. Ştiinţ. Univ. Al I Cuza Iaşi Biol. Veg..

[99] Vochiţa G., Ionescu E., Tebrencu C.E., Ciupercă O.T., Mihai C.T., Gherghel D. In Vitro Investigation of Antitumoral Activity Mechanisms of *Ulmus glabra* Bark Extracts. Proceedings of the 11th National Congress with International Participation and the 37th Annual Scientific Session of the Romanian Society of Cell Biology.

[100] Ciupercă O.T., Ţebrencu C.E., Ionescu E., Iacob E., Volf I. (2019). Studies on Polyphenols Isolated from Branches of *Prunus spinosa* L. Species. Rev. Chim..

[101] Ţebrencu C.E., Ciupercă O.T., Ionescu E. (2020). New Sources of Condensed Tannins—Investigation of Branches of Some Shrub Species Through HPTLC Analysis. Ann. Acad. Rom. Sci. Phys. Chem. Sci..

[102] Gegiu G., Branza A.-D., Bucur L., Grigorian M., Tache T., Badea V. (2015). Contributions to the Antimicrobial and Antifungal Study of the Aqueous Extract of *Prunus spinosa* L.. Farmacia.

[103] Veličković I., Žižak Ž., Rajčević N., Ivanov M., Soković M., Marin P.D., Grujić S. (2021). *Prunus spinosa* L. Leaf Extracts: Polyphenol Profile and Bioactivities. Not. Bot. Horti Agrobot. Cluj-Napoca.

[104] Kiran S.R., Deepika D.S., Babu Y.T.R., Chowdary M.R., Kumar G.V. (2024). Evaluation of Antioxidant Potential and Selective Cytotoxicity of Endangered Medicinal Plant *Prunus ceylanica* (Wight) Miq. Ann. Biol..

[105] Hwang D., Kim H., Shin H., Jeong H., Kim J., Kim D. Cosmetic Effects of *Prunus padus* Bark Extract. Korean J. Chem. Eng..

